# Hidden three-state survival model for bivariate longitudinal count data

**DOI:** 10.1007/s10985-018-9448-1

**Published:** 2018-08-27

**Authors:** Ardo van den Hout, Graciela Muniz-Terrera

**Affiliations:** 1 Department of Statistical Science, University College London, London, UK; 2 Centre for Dementia Prevention, University of Edinburgh, Edinburgh, UK

**Keywords:** Bivariate binomial distribution, Markov model, Cognitive function, Stochastic process, Latent-class model

## Abstract

A model is presented that describes bivariate longitudinal count data by conditioning on a progressive illness-death process where the two living states are latent. The illness-death process is modelled in continuous time, and the count data are described by a bivariate extension of the binomial distribution. The bivariate distributions for the count data approach include the correlation between two responses even after conditioning on the state. An illustrative data analysis is discussed, where the bivariate data consist of scores on two cognitive tests, and the latent states represent two stages of underlying cognitive function. By including a death state, possible association between cognitive function and the risk of death is accounted for.

## Introduction

In ageing research, longitudinal data are often collected for various tests of cognitive function. For example, the English Longitudinal Study of Ageing (ELSA) collects data on literacy, numeracy, memory, and information processing (Huppert et al. 2006). In ELSA and often in other studies, the range for test scores is in the form of counts; that is, $\{0,1,..,m\}$, where *m* is a test-specific integer. Important questions in ageing research concern individual change in cognitive function in the population. When longitudinal data are used, death during follow-up cannot be ignored in the statistical analysis because the risk of death is likely to be correlated with change in cognitive function (Muniz-Terrera et al. 2011).

Typically, cognitive function is seen as a latent trait that explains observed test scores. Of specific interest is to detect when a change in the observed scores is indicative of an irreversible decline in cognitive function. When two tests are available, it make sense to look at options to model both tests simultaneously. For this reason, we propose a statistical framework which allows inference on change in cognitive function by modelling longitudinal count data for two correlated tests conditional on two latent states of cognitive function, and with death as an observable third state.

The three-state process is modelled in continuous time using the Markov assumption. Because the two living states are latent, the three-state model is called a *hidden Markov model*; see also, for example, Baum and Petrie (1966), Satten and Longini (1996), Jackson (2011), and Zucchini et al. (2016). The count data are conditionally modelled by the bivariate binomial distribution as introduced by Altham and Hankin (2012). The approach is general and can also be used in other areas of applied statistics. For example, if longitudinal count data are collected on biomarkers, and dropout during the follow-up is correlated with the biomarkers, then the same methodology can be used.

Our hidden Markov model can be seen as an alternative for a joint-model approach where a model for a longitudinal response is combined with a two-state survival model (alive versus death); see, for example, Rizopoulos (2012). Using two latent living states in addition to the death state allows us to describe the data in a way which is very close to our understanding of cognitive function as a latent trait. By making the three-state model progressive, we model the assumption that cognitive decline is an irreversible process. In addition, we can use a fitted model to predict latent cognitive impairment without specifying thresholds on the scale of cognitive tests. This will be illustrated in the application.

Examples of papers that discuss change of cognitive function conditional on a multi-state model are Dantan et al. (2011), Van den Hout et al. (2015), and Rouanet et al. (2016). These papers combine a multi-state model with a model for a univariate test (or latent trait) for cognitive function. Our statistical framework is an extension of this approach by introducing a bivariate state-dependent distribution. This allows us to model two tests for cognitive function simultaneously.

Our hidden Markov model is similar in aim and structure to the modelling in Cook et al. (2004). Cook et al. discuss hidden Markov models where dependence in multivariate tests is taken into account by discrete distributions through log-linear models. They combine a discrete-time two-state Markov model with log-linear models for a bivariate binary response variable. Our model differs in two important aspects. First, we specify a continuous-time Markov model so that we can deal with interval-censored data and irregular follow-up times. Second, our model is specified for count data which motivates another model for the bivariate state-dependent distributions.

For time $t\ge 0$, let $Y_t \in \{0,1,.., m_Y\}$ and $Z_t\in \{0,1,.., m_Z\}$ denote bivariate longitudinal count data, and let $X_t\in \{1,2,3\}$ denote a three-state progressive survival process where state 3 indicates death.

For $Y_t$ and $Z_t$, data are collected at pre-scheduled times; that is, the longitudinal sampling is designed independently from $Y_t$ and $Z_t$. This creates interval-censored data in the sense that we do not observed values of $Y_t$ and $Z_t$ between two scheduled time points. This kind of data is also called panel data. We assume that the time of death is observed exactly.

We explain the conditioning on the three-state progressive process by an example. The three-state process $X_t$ is defined by transitions $1\rightarrow 2$, $1\rightarrow 3$, and $2\rightarrow 3$. Assume data $(y_1,y_2)$ and $(z_1,z_2)$ are observed at times $(t_1,t_2)$. Let *p*() be the generic notation of a probability mass function. It follows that

1 $$\begin{aligned} p(y_1,y_2,z_1,z_2)=\sum _{(x_1,x_2)\in \{(1,1),(1,2),(2,2)\}} p(y_1,y_2,z_1,z_2|x_1,x_2)p(x_2|x_1)p(x_1).\nonumber \\ \end{aligned}$$

The summation in (1) is defined for all possible latent-state combinations for $(t_1,t_2)$ taking into account that the process is progressive. Because count data are observed at $t_1$ and $t_2$, we know that at these times the individual is alive.

We simplify (1) by assuming condition independence across time in the sense that

2 $$\begin{aligned} p(y_1,y_2,z_1,z_2|x_1,x_2)=p(y_1,z_1|x_1)p(y_2,z_2|x_2) \end{aligned}$$

Equations (1) and (2) illustrate the main modelling approach. It will be comprised of three parts: state-dependent bivariate binomial distributions for $p(y_j,z_j|x_j)$, for $j=1,2$, an illness-death model for transition probabilities $p(x_2|x_1)$, and a logistic regression model for the initial distribution $p(x_1)$.

We could go one step further and assume independence between $Y_t$ and $Z_t$ conditional on latent state. This would imply $p(y_j,z_j|x_j)=p(y_j|x_j)p(z_j|x_j)$, for $j=1,2$. This assumption underlies some of the hidden multi-state models that are discussed in the literature; see, for example, Jackson et al. (2016). This will be explored in the data analysis as a comparison to the bivariate approach.

## Model

This section introduces the three submodels that define the encompassing hidden Markov model for the bivariate longitudinal count data. The first model is the bivariate binomial distribution of the count data conditional on the latent state. The second model is the stochastic Markov process for the two latent states and death. The third model is the distribution of the initial state.

### Bivariate binomial distribution

Altham (1978) introduced the following extension of the standard binomial distribution

3 $$\begin{aligned} p(Y=y)=\frac{{{m}\atopwithdelims (){y}} p^y (1-p)^{(m-y)}\theta ^{y(m-y)}}{g(p,\theta ,m)} \quad \text{ for }\quad y\in \{0,1,...,m\}, \end{aligned}$$

where $$0< p <1$$, $$\theta >0$$, and

$$\begin{aligned} g(p,\theta ,m) = \sum _{y=0}^m {{m}\atopwithdelims (){y}} p^y (1-p)^{(m-y)}\theta ^{y(m-y)}. \end{aligned}$$

For $\theta > 1$ or $\theta <1$, the distribution defines *under*- or *over*dispersion, respectively, relative to the standard binomial distribution.

The bivariate version of (3) is presented in Altham and Hankin (2012) and is given by

4 $$\begin{aligned} p(Y=y,Z=z)=\frac{{{m}\atopwithdelims (){y}} p_Y^y (1-p_Y)^{(m-y)}\theta _Y^{y(m-y)} {{m}\atopwithdelims (){z}} p_Z^z (1-p_Z)^{(m-z)}\theta _Z^{z(m-z)}\phi ^{yz}}{C},\nonumber \\ \end{aligned}$$

for $y,z\in \{0,1,...,m\}$, where $0< p_Y,p_Z <1$, $\theta _Y,\theta _Z,\phi >0$, and

$$\begin{aligned} C = \sum _{y=0}^m \sum _{z=0}^m {{m}\atopwithdelims (){y}} p_Y^y (1-p_Y)^{(m-y)}\theta _Y^{y(m-y)} {{m}\atopwithdelims (){z}} p_Z^z (1-p_Z)^{(m-z)}\theta _Z^{z(m-z)}\phi ^{yz}. \end{aligned}$$

It is possible to define this distribution for the case where the sample space for *Y* and *Z* is not the same (replace the corresponding *m* by $m_Y$ and $m_Z$).

For the three-state model in the next section, we will specify two bivariate binomials by conditioning on the two living states; that is, $p(Y,Z|X=1)$ and $p(Y,Z|X=2)$. To manage the number of parameters, restrictions can be defined across these two distributions. In the data analysis in Sect. 4, for example, we assume that the correlation parameter $\phi$ is the same for the two distributions.

### Progressive illness-death process

Methods for multi-state models for longitudinal data are well established; see, for example, Hougaard (1999) and Aalen et al. (2008). For the case where transition times are interval-censored, see, for example, Kalbfleisch and Lawless (1985) and Jackson (2011). The following follows the presentation in Van den Hout (2017), who describes parametric time-dependent hazard models for interval-censored data.

Let $q_{rs}(t)$ denote the hazard for transition $r\rightarrow s$ at time *t*. Given time interval $(t_1,t_2]$, the cumulative hazard functions for leaving state 1 and 2 are given by

$$\begin{aligned} H_1(t_1,t_2)=\int _{t_1}^{t_2}q_{12}(u)+q_{13}(u)du \quad \text{ and } \quad H_2(t_1,t_2)=\int _{t_1}^{t_2}q_{23}(u)du, \end{aligned}$$

respectively. Given that state 3 is an absorbing state and that the model is progressive, if follows that $q_{21}(t)=q_{31}(t)=q_{32}(t)=0$. Transition probabilities $p_{rs}(t_1,t_2)=P\left( X_{t_2}=s|X_{t_1}=r\right)$ are given by

$$\begin{aligned} p_{11}(t_1,t_2)= & {} \exp \big (-H_1(t_1,t_2)\big )\\ p_{12}(t_1,t_2)= & {} \int _{t_{1}}^{t_2}\exp \big (-H_1(t_1,u)\big )q_{12}(u)\exp \big (-H_2(u,t_2)\big )du\\ p_{13}(t_1,t_2)= & {} 1-p_{11}(t_1,t_2)-p_{12}(t_1,t_2)\\ p_{21}(t_1,t_2)= & {} 0\qquad p_{31}(t_1,t_2)\ = \ 0\\ p_{22}(t_1,t_2)= & {} \exp \big (-H_2(t_1,t_2)\big )\, \qquad \; p_{32}(t_1,t_2)\ =\ 0\\ p_{23}(t_1,t_2)= & {} 1-p_{22} \qquad p_{33}(t_1,t_2)\ =\ 1 \,. \end{aligned}$$

Examples of hazard models for $(r,s)\in \{(1,2),(1,3),(2,3)\}$ are

$$\begin{aligned} \begin{array}{lll} \text {Exponential model: }&{} \quad q_{rs}(t)=\lambda _{rs} &{}\quad \lambda _{rs}>0\\ \text {Gompertz model: } &{}\quad q_{rs}(t)=\lambda _{rs}\exp (\xi _{rs} t) &{}\quad \lambda _{rs}>0\\ \text {Weibull model: } &{}\quad q_{rs}(t)=\lambda _{rs}\tau _{rs} t^{\tau _{rs}-1} &{}\quad \lambda _{rs},\tau _{rs}>0\, . \end{array} \end{aligned}$$

The framework is very flexible in the sense that the choice of parametric models can vary across the transitions. For example, a hazard model can be defined that is exponential for $1\rightarrow 3$ and $2\rightarrow 3$, and Gompertz for $1\rightarrow 2$. Alternatively, we can define a hazard model that is Gompertz for $1\rightarrow 3$ and $2\rightarrow 3$, and Weibull for $1\rightarrow 2$.

For the exponential model, transition probabilities $p_{rs}(t_1,t_2)$ can be derived in closed form. For the time-dependent Gompertz and Weibull models, or for combinations thereof, probabilities $p_{12}(t_1,t_2)$ are computed by numerical approximation of the one-dimensional integrals; for the details see the appendix.

It is possible to fit the time-dependent models by using a piecewise-constant approximation for the hazards. For example, the Gompertz model can be fitted in the R-package msm by approximating $q_{rs}(u)$ for $u\in (t_1,t_2]$ by $q_{rs}(t_1)$; see Jackson (2011). In what follows, we do not use this piecewise-constant approximation.

The parametric hazard models can be extended in a standard way to include effects of covariates. As an example, if we would like to investigate the effect of covariate *w*, then we can specify $q_{rs}(t)=\lambda _{rs}\exp (\xi _{rs}t)\exp (\beta _{rs}w)$.

### Initial distribution

As shown in the introduction, a model is needed to estimate the state prevalence; that is, the initial distribution $p(x_t)$ for time $t>0$ and $x_t\in \{1,2\}$. We use the standard logistic regression model

5 $$\begin{aligned} p(X_t=2)=\frac{\exp (\eta _0+\eta _1 t)}{1+\exp (\eta _0+\eta _1 t)}. \end{aligned}$$

and define $p(X_t=1)=1-p(X_t=2)$. Covariates can be included in the usual way.

## Likelihood function and estimation

Estimation of model parameters is undertaken by maximising the log-likelihood function. Let $i=1,...,N$ denote individuals, and $t_{ij}$ the time of the *j*th observation for individual *i*, where $j=1,...,n_i$. The count data at time $t_{ij}$ are given by pairs $(y_{ij},z_{ij})$. Let $\mathbf {y}_i=(y_{i1},...,y_{in_i})$, and $\mathbf {y}=(\mathbf {y}_{1},...,\mathbf {y}_{N})$. Define $\mathbf {z}$ in a similar manner. Let $x_{ij}$ denote the state at $t_{ij}$, which is latent except when death is observed.

Conditional on the effect of time *t* and possible covariates, we assume that the three-state process is Markovian; that is, only the current state determines where the process goes next. This allows us the decompose the probability mass function for a series of states into univariate transition probabilities. For example, $p(x_{i3},x_{i2}|x_{i1})$ is assumed to be equal to $p(x_{i3}|x_{i2})p(x_{i2}|x_{i1})$.

Let vector ${\varvec{\psi }}$ contain all model parameters. Using the conditional Markov assumption and the assumption about independence across time (see the introduction), the likelihood function is given by

$$\begin{aligned} L({\varvec{\psi }}|\mathbf {y},\mathbf {z})= & {} \prod _{i=1}^N \sum _{\mathbf {x}_i\in \varOmega _{i}} L_{n_i}\times p(y_{in_i-1},z_{in_i-1}|x_{in_i-2})\times \cdot \cdot \cdot \times p(y_{i1},z_{i1}|x_{i1})\\&\times K_{n_i}\times p(x_{in_i-1}|x_{in_i-2})\times \cdot \cdot \cdot \times p(x_{i2}|x_{i1})\times p(x_{i1}),\\ \end{aligned}$$

where

$$\begin{aligned} \left. \begin{array}{l} L_{n_i}=p(y_{in_i},z_{in_i}|x_{in_i})\\ K_{n_i}= p(x_{in_i}|x_{in_i-1})\\ \varOmega _{i} =\big \{\text {all possible } \mathbf {x}_i=(x_{i1},...,x_{in_i})\big |x_{in_i}\in \{1,2\}\big \} \end{array} \right\} \quad \text {if alive at }t_{in_i}, \end{aligned}$$

and

$$\begin{aligned} \left. \begin{array}{l} L_{n_i}=1\\ K_{n_i}= \mathop {\sum }\nolimits _{s=1}^2 p(X_{in_i}=s|x_{in_i-1})q_{s3}(t_{in_i})\\ \varOmega _{i} =\big \{\text {all possible }\mathbf {x}_i=(x_{i1},...,x_{in_i-1})\big \} \end{array} \right\} \quad \text {if death occurs at }t_{in_i}\,. \end{aligned}$$

The logarithm of the likelihood function is maximised over the parameter space of ${\varvec{\psi }}$ to obtain the maximum likelihood estimates. The maximisation is undertaken using the general-purpose optimiser optim in the R software (R Core Team 2013). In optim, we choose the quasi-Newton method BFGS (developed in 1970 by Broyden, Fletcher, Goldfarb and Shanno). The numerical integration needed to compute probabilities $p_{12}(t_1,t_2)$ is implemented using the composite Simpson’s rule.

For efficient use of the general-purpose optimiser, all parameters with restricted parameters space are transformed such that they can be estimated over an unrestricted parameter space. For example, if $0< \psi <1$, then define $\psi =\exp (\gamma )/(1+\exp (\gamma ))$ and estimate $\psi$ by estimating $\gamma \in \mathbb {R}$. The delta-method may be used to estimate the variance of $\widehat{\psi }$ given estimated variance of $\widehat{\gamma }$. Alternatively, simulation may be used by sampling from the asymptotic normal distribution of the maximum likelihood estimate; see Mandel (2013). For the above example this implies sampling values of $\widehat{\gamma }$ and use these to derive the distribution of $\widehat{\psi }$.

For the latent living states we parameterise the binomial distributions such that state 2 is the state with a lower cognitive ability. We illustrate this for the bivariate distribution $p(Y,Z|X=x)$, where $x\in \{1,2\}$. First we parameterise $p_{Y|x=1}=\exp (\gamma )/(1+\exp (\gamma ))$ for $\gamma \in \mathbb {R}$, so that $0<p_{Y|x=1}<1$. Next we define

6 $$\begin{aligned} p_{Y|x=2}=\frac{\exp (\gamma -\exp (\nu ))}{1+\exp (\gamma -\exp (\nu ))} \, , \end{aligned}$$

for $\nu \in \mathbb {R}$. This implies $p_{Y|x=1}>p_{Y|x=2}>0$. We do the same for $p_{Z|x=1}$ and $p_{Z|x=2}$.

## Analysis

### Data

The English Longitudinal Study of Ageing (ELSA) is a multidisciplinary open cohort study that features an extensive range of data from a representative sample of men and women living in England who are aged 50 and over. Here we use longitudinal data on cognitive function that are available in the waves 1-5 (2002-2011). Data from ELSA can be obtained via the Economic and Social Data Service (www.esds.ac.uk). For reasons of confidentiality, age is given in integers.

There are 11,932 individuals in the ELSA baseline sample. For the current analysis, we sampled $N=1000$ individuals randomly from the baseline conditional on three restrictions. First, we restricted the sampling to individuals being 50 years or older at the ELSA baseline (because participants’ partners are also sampled in ELSA, there are individuals younger than 50 in the ELSA baseline). Secondly, the sampling is restricted to individual younger than 90 years at baseline without a censored age during follow-up. Thirdly, the sampling is restricted to those who have at least two observations or one observation and a time of death. The resulting subsample has 540 women and 460 men. Frequencies for number of observations per individual (including death) are 174, 146, 156, 516, and 8 for number of observations 2, 3, 4, 5, and 6, respectively. The data are used for illustrative purposes only and should not be used to inform clinical practice.

We analyse longitudinal count data for the test of verbal learning and recall. For this test, people are required to learn a list of 10 common words, and are asked to recall the words immediately (*Y*) and later on in the interview (*Z*). This is a common test in studies of ageing. For example, it is also used the US Health and Retirement Study (website: hrsonline.isr.umich.edu).

**Fig. 1:**
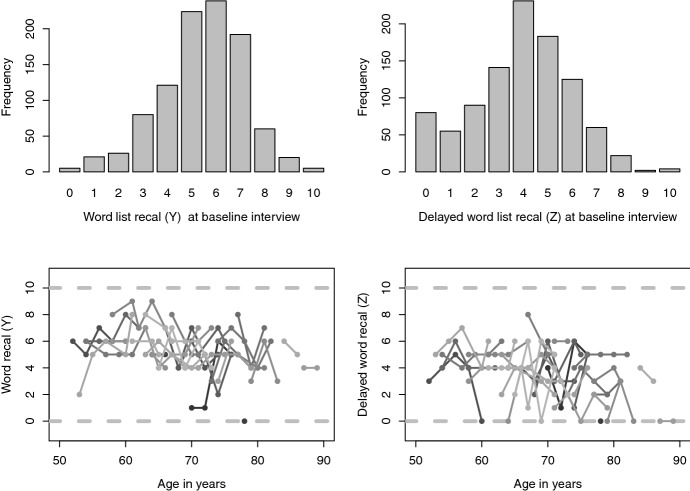
ELSA count data. Bottom for a random subset of 30 individuals

As to be expected, individuals remember more words in the direct recall compared to the delayed recall. The top panel of Fig. 1 shows the sample distribution of *Y* and *Z* at the baseline. The distribution clearly shifts to the left, from the sample mean 5.44 for *Y* to the sample mean 3.92 for *Z*. One would expect a high correlation between *Y* and *Z* given that these concern the recall of the same words, and this is confirmed by the Spearman correlation which is 0.67 for the observations at baseline.

The bottom panel of Fig. 1 shows the longitudinal scores on *Y* and *Z* for a subsample of 30 individuals. The 30 trajectories illustrate that the word recalls are noisy processes. Nevertheless, already in the depicted trajectories there is some evidence of a decline in cognitive function when people get older.

During the follow-up, 195 of the 1000 individuals die. A dropout rate of 19.5% is too high to ignore in the data analysis, especially if the process of interest is associated with ageing. This motivates the inclusion of a death state in the stochastic process.

### Models

We will discuss a series of models for the ELSA data. The information criterion by Akaike (AIC, Akaike 1974) is used to compare the models. Given that ELSA is an epidemiological study on ageing, we choose age as the time scale.

In all models, the initial distribution for the two living states is described by the logistic regression (5) which includes age as a covariate. For the state-dependent distribution we distinguish three options: two independent binomial distributions, two independent extended binomial distributions (3), and the bivariate extended binomial distribution (4).

For the three-state model, we consider exponential ($\mathcal {E}$), Gompertz ($\mathcal {G}$), and Weibull ($\mathcal {W}$) hazards. The models are denoted by using the letters for the transitions $1\rightarrow 2$, $1\rightarrow 3$, and $2\rightarrow 3$ respectively. For example, $\mathcal {G}\mathcal {W}\mathcal {W}$ denotes a Gompertz hazard for $1\rightarrow 2$, and Weibull hazards for death.

For the exponential model $\mathcal {E}\mathcal {E}\mathcal {E}$, it is clear that the bivariate binomial distribution outperforms the other two options for the state-dependent distribution; see the AICs in Table 1. This is as expected, given the correlation between the immediate recall and the delayed recall.

**Table 1:** AIC, number of parameters, and minus twice the maximum value of the log-likelihood function (-2LL) of the various models fitted to the ELSA data

Hazard distributions	Initial distribution	Binomial distribution(s)	#Parameters	-2LL	AIC
$\mathcal {E}\mathcal {E}\mathcal {E}$	Age	Standard univariate	9	31124	31142
$\mathcal {E}\mathcal {E}\mathcal {E}$	Age	Extended univariate	11	31026	31048
$\mathcal {E}\mathcal {E}\mathcal {E}$	Age	Bivariate	12	29708	29732
$\mathcal {G}\mathcal {G}\mathcal {G}$	Age	Bivariate	15	29616	29646
$\mathcal {G}\mathcal {G}\mathcal {G} + \text {gender}$	Age	Bivariate	18	29603	29639
$\mathcal {G}\mathcal {G}\mathcal {G} + \text {gender}$	Age $+$ gender	Bivariate	19	29598	29636
$\mathcal {W}\mathcal {W}\mathcal {W}$	Age	Bivariate	15	29621	29651
$\mathcal {G}\mathcal {W}\mathcal {W}$	Age	Bivariate	15	29619	29649
$\mathcal {G}\mathcal {W}\mathcal {W}+ \text {gender}$	Age	Bivariate	18	29611	29647
$\mathcal {G}\mathcal {W}\mathcal {W}+ \text {gender}$	Age $+$ gender	Bivariate	19	29606	29644
$\mathcal {W}\mathcal {G}\mathcal {G}$	Age	Bivariate	15	29688	29718

Letters for the hazard models ($\mathcal {E}$xponential, $\mathcal {G}$ompertz, and $\mathcal {W}$eibull) for $1\rightarrow 2$, $1\rightarrow 3$, and $2\rightarrow 3$, respectively

Given that decline of cognitive function is correlated with age, one would expect that time-dependent hazards models are better than the exponential hazard model. This is confirmed by the results in Table 1. For the bivariate binomial distribution, using the Gompertz model $\mathcal {G}\mathcal {G}\mathcal {G}$ yields a substantial lower AIC compared to $\mathcal {E}\mathcal {E}\mathcal {E}$: AIC = 29646 and AIC = 29732, respectively. The same holds for the Weibull model $\mathcal {W}\mathcal {W}\mathcal {W}$ with AIC = 29651.

As reported in Table 1, we investigated a range of models, including models with different parameter assumptions for the three hazards. The lowest AIC is 29636 for a model with three Gompertz hazards ($\mathcal {G}\mathcal {G}\mathcal {G}$). This model has age (denoted by *t*) and gender (denoted by $x=0$ for women, and $x=1$ for men) as predictors for the initial distribution; that is,

$$\begin{aligned} p(X_t=2)=\frac{\exp (\eta _0+\eta _1 t +\eta _2 x)}{1+\exp (\eta _0+\eta _1 t+\eta _2 x)}. \end{aligned}$$

The three Gompertz hazards are extended by including gender as covariate:

$$\begin{aligned} h_{rs}(t)=\exp (\beta _{rs}+\xi _{rs}t+\alpha _{rs} x)\quad \text{ for }\quad (r,s)\in \{(1,2),(1,3),(2,3)\}. \end{aligned}$$

The state-dependent distributions are bivariate binomials, where the dispersion parameters $\theta _Y$ and $\theta _Z$, and the correlation parameter $\phi$ are assumed to be the same for the two latent states. This overall model has 19 parameters; Table 2 presents the estimates and their estimated standard errors.

**Table 2:** Estimated model parameters for the Gompertz hazard model $\mathcal {G}\mathcal {G}\mathcal {G}$, the logistic regression for the initial distribution, and the bivariate binomial state-dependent distribution

Parameter	Estimate		Parameter	Estimate	
$\beta _{12}$	$-$ 7.554	(1.494)	$p_{Y|x=1}$	0.310	(0.012)
$\beta _{13}$	$-$ 6.169	(0.488)	$p_{Z|x=1}$	0.118	(0.007)
$\beta _{23}$	$-$ 6.045	(0.482)	$p_{Y|x=2}$	0.219	(0.008)
$\xi _{12}$	0.149	(0.062)	$p_{Z|x=2}$	0.063	(0.005)
$\xi _{13}$	0.096	(0.023)	$\theta _Y$	1.138	(0.009)
$\xi _{23}$	0.103	(0.015)	$\theta _Z$	1.040	(0.007)
$\alpha _{12}$	$-$ 0.502	(1.347)	$\phi$	1.391	(0.015)
$\alpha _{13}$	$-$ 0.035	(0.363)	$\eta _{0}$	$-$ 3.318	(0.293)
$\alpha _{23}$	0.527	(0.176)	$\eta _{1}$	0.166	(0.014)
			$\eta _{2}$	0.463	(0.219)

Estimated standard errors within brackets

As expected, the effects of age (the $\xi$s) are positive and indicate that an older age is associated with a higher risk of progressing through the states. For the three transition hazards, only the estimated hazard for $2\rightarrow 3$ indicates a difference between men and women, with the former being at a higher risk (hazard ratio $\exp (\alpha _{23})=1.694$).

The parameters for the state-dependent distributions for the direct recall (*Y*) and the delayed recall (*Z*) are estimated using transformations, among which the transformation (6). Table 2 presents the parameters as used in the definition of the distributions and with estimated standard errors derived by simulation (see Sect. 3). Given that $\theta _Y$ and $\theta _Z$ are both larger than 1, the state distributions are more peaked than the standard binomial. Parameter $\phi$ controls the dependence between the distributions of *Y* and *Z* within each state, with *Y* and *Z* being independent iff $\phi =1$. Although more can be said about the interpretation of $\phi$ (see Altham and Hankin 2012), in practice the best option is to look at graphs and at the estimated means.

**Fig. 2:**
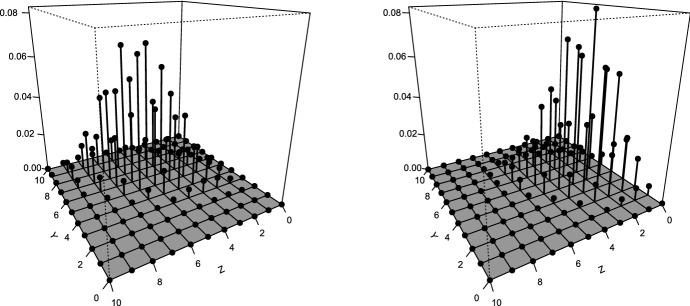
Fitted state-dependent bivariate binomial distributions for *Y* (direct recall) and *Z* (delayed recall). Left-hand side: conditional on state 1. Right-hand side: conditional on state 2

Figure 2 nicely shows the difference in the fitted state-dependent bivariate binomial distributions for *Y* and *Z*. Comparing the distributions for state 1 and 2, we see that the distribution for state 2 is shifted to the lower scores on *Y* and *Z*. Numerically this is confirmed by the expected value of the fitted distributions. For state 1, we have $$E[(Y,Z)|X= 1] = (6.37, 5.19) $$, and for state 2 we have $$E[(Y,Z)|X=2] = (4.36, 2.60)$$.

**Fig. 3:**
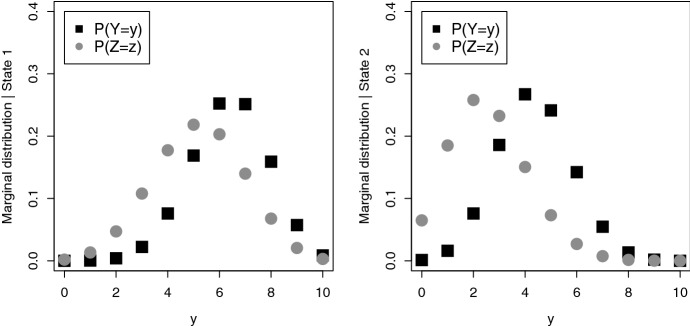
Fitted marginal state-dependent bivariate binomial distributions for *Y* (direct recall) and *Z* (delayed recall). Left-hand side: conditional on state 1. Right-hand side: conditional on state 2

The shift to the lower scores is additionally illustrated by the marginal distributions in Fig. 3. This graph also shows that the marginal distributions of the delayed recall (*Z*) are more distinct than those of the direct recall (*Y*). This implies that the delayed recall is better at discriminating the two latent cognitive states.

### Prediction

Of specific interest is the ability to infer on the latent state given observed scores. Let $\mathbf {y}_i=(y_{i1},...,y_{in_i})$ and $\mathbf {z}_i=(z_{i1},...,z_{in_i})$ be the observed count data for individual *i* at times $(t_{i1},...,t_{in_i})$. To predict the latent state at $t\ge t_{in_i}$, we can compute

$$\begin{aligned}&p(X_t=1|\mathbf {y}_i, \mathbf {z}_i,{\varvec{\psi }}=\widehat{{\varvec{\psi }}})= \sum _{\mathbf {x}_i\in \varOmega _{i}}p(X_t=1|\mathbf {x}_i,\mathbf {y}_i, \mathbf {z}_i,{\varvec{\psi }}=\widehat{{\varvec{\psi }}}) p(\mathbf {x}_i|\mathbf {y}_i, \mathbf {z}_i,{\varvec{\psi }}=\widehat{{\varvec{\psi }}})\\&\quad =\sum _{\mathbf {x}_i\in \varOmega _{i}}p(X_t=1|\mathbf {x}_i,{\varvec{\psi }}=\widehat{{\varvec{\psi }}}) \frac{p(\mathbf {y}_i, \mathbf {z}_i|\mathbf {x}_i,{\varvec{\psi }}=\widehat{{\varvec{\psi }}})p(\mathbf {x}_i|{\varvec{\psi }}=\widehat{{\varvec{\psi }}})}{\sum _{\mathbf {x}^*_i\in \varOmega _{i}}p(\mathbf {y}_i, \mathbf {z}_i|\mathbf {x}^*_i,{\varvec{\psi }}=\widehat{{\varvec{\psi }}}) p(\mathbf {x}^*_i|{\varvec{\psi }}=\widehat{{\varvec{\psi }}})}, \end{aligned}$$

where vector $\widehat{{\varvec{\psi }}}$ contains the estimated model parameters, and $\varOmega _{i} =\big \{\text {all possible } \mathbf {x}_i=(x_{i1},...,x_{in_i})\big \}$.

We illustrate this prediction for three individuals (*A*, *B*, and *C*, say) for whom we assume that we have yearly test scores at age 70 up to 74. The scores are made up and are given by the following (*y*, *z*)-pairs for the successive years:

$$\begin{aligned}&\text {for }A: (8,5),(7,6),(8,5),(7,6),(8,5),\\&\text {for }B: (7,4),(6,3),(7,4),(6,3),(5,4),\\&\text {for }C: (7,6),(6,5),(5,4),(4,3),(3,2). \end{aligned}$$

These scores were specified such that *A* represents an individual with very good recall, *C* represent an individual whose scores show a decreasing trend, and individual *B* is more or less in the middle of these two trends.

**Fig. 4:**
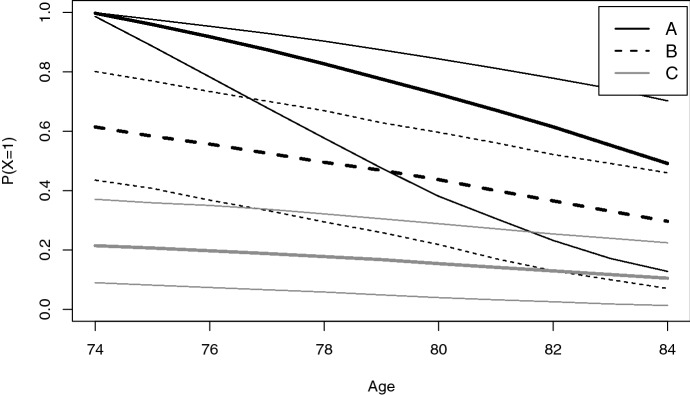
Predicted probability to be in latent state 1 for three individuals. Prediction is based on the fitted model and specified yearly test scores at age 70 up to 74; see Sect. 4.3. Thin lines for 90% confidence bands

Figure 4 shows the predicted probability to be in state 1 for the three individuals. Note that death is a competing risk in this prediction, so it is *not* the case that $P(X_t=1)=1-P(X_t=2)$, for *t* representing a higher age than 74.

The graph shows that we can classify individuals into the latent states given past observations. For example, at age 74 and using 0.5 as cut-point, we would classify *A* and *B* in state 1, and *C* in state 2. The graph also shows that the model can be used in practice to plan future health care. For individual *B*, for example, the model predicts a classification in state 2 from about age 79 and onward (using again 0.5 as cut-point). If there are interventions to delay cognitive decline, then for *B* such an intervention should be planned in the next five years.

The 90% confidence bands in Fig. 4 show that a classification such as above is subject to substantial uncertainty. In general, adding covariates in the submodels may help to reduce this uncertainty up to some extent.

### Validation

As acknowledged in the literature, model validation for time-dependent multi-state processes is severely hampered when transitions are interval-censored and observation times vary across individuals; see, for example, Gentleman et al. (1994) and Titman (2009). This problem also holds for the models reported here where we use a three-state process which is latent for the two living states. However, observed death times are exact, and this can be used to check part of the model. The idea is to fit Kaplan-Meier survival curves where the time scale is the study time and death is defined as the event. These curves are then compared to the model-based prediction conditional on the observed data at the baseline of the study. This will not validate all aspects of the model, but it can be used as a heuristic tool to check observed and predicted survival (Gentleman et al. 1994).

The method in the previous section can be used to undertake the model-based prediction. Denote the time scale of the study in years as $t^*$, with $t^*=0$ at the baseline. Using the same notation as before, let $y_{i1}$ and $z_{i1}$ be the observed count data at $t^*=0$, for all $i\in \{1,...,N\}$. The model-based survival at time $t^*>0$ is

$$\begin{aligned} p(X_{t^*}=3|{y}_{i1}, {z}_{i1},{\varvec{\psi }}=\widehat{{\varvec{\psi }}}), \end{aligned}$$

which is to be compared with the Kaplan-Meier survival curves. To do this, we first sample the latent baseline state at $t^*=0$ using $p(X_{t^*=0}=1|{y}_{i1}, {z}_{i1},{\varvec{\psi }}=\widehat{{\varvec{\psi }}})$ for all $i=1,..,N$. Next we compute the Kaplan-Meier survival curves and the model-based survival conditional on the sampled baseline state.

**Fig. 5:**
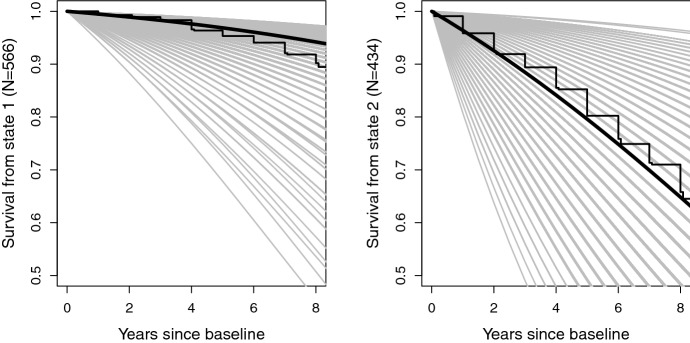
Comparison of Kaplan-Meier survival curves (step function) and model-based predicted survival conditional on baseline score with the thick line for the median of the model-based individual curves (grey lines)

Figure 5 shows the comparison. Variation in the model-based individual curves is due to individual variation at baseline with respect to age, gender, and the scores on *Y* and *Z*. The regular yearly steps in the Kaplan-Meier survival curves are caused by the fact that age is only available in integers for reasons of confidentiality.

Given that only baseline test scores are used, and that the baseline state is latent, the comparisons in Fig. 5 give some confidence in the fitted model. At least with respect to fitting observed and right-censored death times, the model performs well.

## Conclusion

In biostatistics, it often the case that the process of interest is latent and that it is investigated via observable response variables. A process of interest in ageing research is change of cognitive function in the older population. Response variables in this case can be scores on cognitive tests. For an application in ageing research with two longitudinal test scores, our approach consists of assuming two latent states for cognitive function and specifying bivariate state-specific distributions for the test scores. In addition, we include death as a third state in our model to take into account possible correlation between change of cognitive function and mortality.

For the response variable, we specify bivariate state-dependent distributions which allow modelling the correlation between two responses even after conditioning on the latent state. In the application, the state-dependent distribution is a bivariate extension of the well-known univariate binomial distribution. In the data analysis, it was shown that a fitted distribution can be explored both numerically and graphically. Goodness of fit and prediction was discussed and illustrated as well.

The underlying progressive three-state model that we use, is very flexible with respect to the specification of the time-dependency. Although the chosen model specifies Gompertz distributions for all three transition hazards, we have also explored hazard models with both Gompertz and Weibull hazards.

The fitting of the three-state model is computationally intensive because some of the transition probabilities are estimated by numerical integration. An alternative would be to use a piecewise-constant approximation of the time-dependent hazards, but the performance of such an approximation would rely heavily on the time grid that is used. Of course, the numerical integration also relies on a grid-based approximation. However, the latter is used to compute one entry in the transition matrix, whereas the former is a piecewise-constant approximation of the whole matrix. Ultimately, both approaches will produce the same results when fine-tuned. We prefer the numerical integration because it is more directly aimed at the quantity that we need to estimate and it is easier to fine tune.

In our hidden Markov model, we assume independence across time and use a bivariate distribution to model possible correlation between response variables. In the current context, where we have longitudinal data clustered within individuals, an alternative would be to use a model with individual-specific random effects. These random effects can be used to capture possible dependence across time or dependence between response variables. For discrete-time hidden Markov models, examples of this approach can be found in Altman (2007) and Maruotti (2011). These mixed hidden Markov models are computationally intensive because the random-effects structure requires numerical integration or simulation-based methods. Continuous-time mixed hidden Markov models can be developed in a similar vein, but will extend the computational challenge.

We have used the bivariate generalisation of the binomial distribution as introduced by Altham and Hankin (2012). Because the fitting the model is undertaken by a general-purpose optimiser, it is relatively easy to use other distributions. For example, other bivariate generalisations of the binomial distribution are presented in Marshall and Olkin (1985) and Biswas and Hwang (2002). The distribution by Altham and Hankin (2012) was chosen not only because it extends the binomial distribution from univariate to bivariate in a natural way, but also because the extension includes parameters for both over-dispersion and under-dispersion relative to the corresponding binomial distribution.

We hope that our approach will help to maximise the use of available data in longitudinal studies, where it is customary to collect data on multiple cognitive tests, and, consequently, improve knowledge about the processes investigated.

## Appendix

Transition probabilities are presented given a specification of the hazards in the three-state progressive model; see also Van den Hout (2017, Chapter 3) for some of the following details.

As before, letters are used to denote the hazard distribution in the order $1\rightarrow 2$, $1\rightarrow 3$, $2\rightarrow 3$. For example, $\mathcal {G}\mathcal {W}\mathcal {W}$ denotes a Gompertz hazard for $1\rightarrow 2$, and Weibull hazards for death.

For the model $\mathcal {G}\mathcal {G}\mathcal {G}$, it follows that

$$\begin{aligned} p_{11}(t_1,t_2)= \exp \Bigg (-\Bigg (\frac{\lambda _{12}}{\xi _{12}}(\exp (\xi _{12}t_2)-\exp (\xi _{12}t_1)) +\frac{\lambda _{13}}{\xi _{13}}(\exp (\xi _{13}t_2)-\exp (\xi _{13}t_1))\Bigg )\Bigg ), \end{aligned}$$

and $p_{22}(t_1,t_2)=\exp (-\frac{\lambda _{23}}{\xi _{23}}(\exp (\xi _{23}t_2)-\exp (\xi _{23}t_1)))$. For $\mathcal {W}\mathcal {W}\mathcal {W}$, we have

$$\begin{aligned} p_{11}(t_1,t_2) = \exp \Big (-\Big (\lambda _{12}(t_2^{\tau _{12}}-t_1^{\tau _{12}})+\lambda _{13}(t_2^{\tau _{13}}-t_1^{\tau _{13}})\Big )\Big ), \end{aligned}$$

and $p_{22}(t_1,t_2)=\exp \left( -\lambda _{23}\left( t_2^{\tau _{23}}-t_1^{\tau _{23}}\right) \right)$. For $\mathcal {W}\mathcal {G}\mathcal {G}$, if follows that

$$\begin{aligned} p_{11}(t_1,t_2) = \exp \Big (-\Big (\lambda _{12}(t_2^{\tau _{12}}-t_1^{\tau _{12}})+\frac{\lambda _{13}}{\xi _{13}}(\exp (\xi _{13}t_2)-\exp (\xi _{13}t_1))\Big )\Big ), \end{aligned}$$

and that $p_{22}(t_1,t_2)$ is the same as for $\mathcal {G}\mathcal {G}\mathcal {G}$. For $\mathcal {G}\mathcal {W}\mathcal {W}$, we get

$$\begin{aligned} p_{11}(t_1,t_2) = \exp \Big (-\Big (\lambda _{13}(t_2^{\tau _{13}}-t_1^{\tau _{13}})+\frac{\lambda _{12}}{\xi _{12}}(\exp (\xi _{12}t_2)-\exp (\xi _{12}t_1))\Big )\Big ), \end{aligned}$$

with $p_{22}(t_1,t_2)$ the same as in $\mathcal {W}\mathcal {W}\mathcal {W}$.

For all the models above, probability $p_{12}(t_1,t_2)$ is derived by a numerical approximation of the integral. Finally, we have $p_{13}(t_1,t_2)=1-p_{11}(t_1,t_2)-p_{12}(t_1,t_2)$ and $p_{23}(t_1,t_2)=1-p_{23}(t_1,t_2)$.
